# Subpopulation-proteomics reveal growth rate, but not cell cycling, as a major impact on protein composition in *Pseudomonas putida* KT2440

**DOI:** 10.1186/s13568-014-0071-6

**Published:** 2014-08-29

**Authors:** Sarah Lieder, Michael Jahn, Jana Seifert, Martin von Bergen, Susann Müller, Ralf Takors

**Affiliations:** 1 Institute for Biochemical Engineering, University of Stuttgart, Allmandring 31, Stuttgart, Germany; 2 Department of Environmental Microbiology, Helmholtz Centre for Environmental Research—UFZ, Permoserstr. 15, Leipzig, 04318, Germany; 3 Department of Proteomics, Helmholtz Centre for Environmental Research—UFZ, Permoserstr. 15, Leipzig, 04318, Germany; 4 Institute of Animal Nutrition, University of Hohenheim, Emil-Wolff-Straße 8 and 10, Stuttgart, 70599, Germany; 5 Department of Metabolomics, Helmholtz Centre for Environmental Research—UFZ, Permoserstr. 15, Leipzig, 04318, Germany; 6 Department of Biotechnology, Chemistry and Environmental Engineering, University of Aalborg, Sohngaardsholmsvej 49, Aalborg, 9000, Denmark

**Keywords:** Heterogeneity, Subpopulations, Pseudomonas putida, Proteome, Flow cytometry, Cell cycle

## Abstract

Population heterogeneity occurring in industrial microbial bioprocesses is regarded as a putative effector causing performance loss in large scale. While the existence of subpopulations is a commonly accepted fact, their appearance and impact on process performance still remains rather unclear. During cell cycling, distinct subpopulations differing in cell division state and DNA content appear which contribute individually to the efficiency of the bioprocess. To identify stressed or impaired subpopulations, we analyzed the interplay of growth rate, cell cycle and phenotypic profile of subpopulations by using flow cytometry and cell sorting in conjunction with mass spectrometry based global proteomics. Adjusting distinct growth rates in chemostats with the model strain *Pseudomonas putida* KT2440, cells were differentiated by DNA content reflecting different cell cycle stages. The proteome of separated subpopulations at given growth rates was found to be highly similar, while different growth rates caused major changes of the protein inventory with respect to e.g. carbon storage, motility, lipid metabolism and the translational machinery.

In conclusion, cells in various cell cycle stages at the same growth rate were found to have similar to identical proteome profiles showing no significant population heterogeneity on the proteome level. In contrast, the growth rate clearly determines the protein composition and therefore the metabolic strategy of the cells.

## Introduction

Commonly applied assumptions consider microbial populations in bioreactors as uniform, thus leveling individual properties of subpopulations to averages. However, it is increasingly accepted that clonal microbial cultures comprise individuals that are not identical, differing in terms of DNA content and cell physiology (Brehm-Stecher and Johnson [2004]; Delvigne and Goffin [2013]). Heterogeneity of clonal microbial cultures may result from several distinct sources, either from internal biological origins, such as mutations, cell cycle decisions and age distribution, or from ‘external’ technical factors (Avery [2006]; Müller et al. [2010]). Notably, external factors interact with biological properties, yielding the superimposition of both impacts in the population. Here, we shed light on the impact of two key players in the origin of population heterogeneity, the growth rate and the cell cycle.

**Figure 1:**
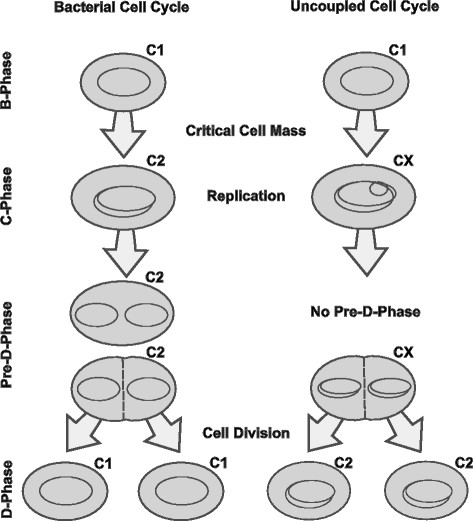
**Schematic overview of the bacterial cell cycle.** The bacterial cell cycle can be divided into B, C, pre-D and D phases constituting a defined order within one generation time. Under unlimited growth conditions, some bacterial species are capable of accelerating proliferation by uncoupling DNA synthesis from division. As a result, a new round of DNA replication is initiated before the completion of the previous round (Cooper [1991]; Müller et al. [2010]).

Traditionally, the cell cycle is suggested to play a role in the development of population heterogeneity within clonal populations (Müller et al. [2010]). A short summary of the sequence of cell cycle phases can be found in Figure 1. The bacterial cell cycle was described for *Escherichia coli* comprising the B-Phase, which is defined as the time between division and start of replication, the replication phase (C-Phase), the pre-D-Phase (an interphase between the C- and D-Phase) and the division phase (D-Phase) (Cooper [1991]; Müller and Babel [2003]). Furthermore, under optimal growth conditions accelerated proliferation (also called ‘multifork DNA-replication’) can be monitored: new rounds of DNA replication may be initiated before a previous round is completed, putatively providing another source of heterogeneity (Bley [1990]; Müller [2007]).

It is suspected, that biosynthesis of biotechnological interesting compounds occurs in dependency of the cell cycle, e.g. only within the stochastic B- and pre-D-phases, when cells are neither replicating nor dividing (Müller et al. [2010]). Ackermann et al. ([1995]) described for *Methylobacterium rhodesianum* that products like polyhydroxyalkanoates (PHAs) accumulate only when cells comprise a certain chromosome number. This phenomenon was found to occur at off-cell-cycling stages. In microbial biotechnology, heterogeneity caused by cell cycling may cause inefficiently producing subpopulations and could have significant impact on the overall process performance (Lencastre Fernandes et al. [2011]). Here, we aim to investigate if the protein inventory of a cell, which is related to its metabolic activity, is dependent on cell cycle stages and how growth rates may influence both, protein composition and cell cycling.

*Pseudomonas putida* KT2440 was used as a model organism owing to its numerous qualities as an expression host, such as safety (Bagdasarian et al. [1981]; Nakazawa and Yokota [1973]), fast growth, a fully sequenced genome (Nelson et al. [2002]) and high stress tolerance (Martins Dos Santos et al. [2004]). Together with simple nutrient demand, the potential to regenerate redox cofactors at a high rate (Blank et al. [2008]) and its amenability to genetic manipulation, *P. putida* is an ideal host for heterologous gene expression (Meijnen et al. [2008]). With the advance of genome-wide pathway modeling (Puchałka et al. [2008]) and ‘omics techniques, the way for systems-wide engineering strategies was paved to turn *P. putida* into a flexible cell factory chassis (Yuste et al. [2006]). Consequently, *P. putida* is more and more explored and already successfully used for numerous industrial applications (Poblete-Castro et al. [2012]; Puchałka et al. [2008]).

In our study, we applied continuous cultivations under controlled growth conditions at defined growth rates. While (fed-) batch approaches are characterized by steadily changing environmental conditions such as media composition, steady-state modes of a chemostat, where cells are cultivated with a pre-installed growth rate, are defined by environmental conditions that remain unchanged (Carlquist et al. [2012]). Notably, (fed-) batch cultures usually represent a mixture of cells growing with different speed as a consequence of changing environmental conditions (Unthan et al. [2014]). Investigating a wide spectrum of growth rates with chemostat cultivation and sampling at steady state conditions gave a specific and unmasked view on the influence of the growth rate on population characteristics. Features like DNA content of the cells, protein composition and adenylate energy charge measurements were included in the study. Additionally, subpopulations with different DNA content were sorted at growth rates 0.1 h^−1^, 0.2 h^−1^ and 0.7 h^−1^ and analyzed for their proteome composition.

Summarizing, we investigated if cell cycling subpopulations at the same growth rate were independent and different from each other on the level of metabolic pathways, e.g. whether slow growing cells with longer cell cycling phases might specialize between proliferation and production phases. In addition, we wanted to clarify if cells invest into different protein species under rising growth rates.

## Materials and methods

### Bacterial strains and cultivation conditions

Chemicals were purchased from Fluka, St. Gallen, Switzerland. Experiments were performed with *P. putida* KT2440 (ATCC 47054) cells originating from a single colony stored in a working cell bank at −70°C. Cells were cultivated in M12 minimal salt medium containing 2.2 gL^−1^ (NH_4_)_2_SO_4_, 0.4 gL^−1^ MgSO_4_ · 7 H_2_O, 0.04 gL^−1^ CaCl_2_ · 2 H_2_O, 0.02 gL^−1^ NaCl, 2 gL^−1^ KH_2_PO_4_ and trace elements (2 mgL^−1^ ZnSO_4_ · 7 H_2_O, 1 mgL^−1^ MnCl_2_ · 4 H_2_O, 15 mgL^−1^ Na_3_-citrate · 2 H_2_O, 1 mgL^−1^ CuSO_4_ · 5 H_2_O, 0.02 mgL^−1^ NiCl_2_ · 6 H_2_O, 0.03 mgL^−1^ NaMoO_4_ · 2 H_2_O, 0.3 mgL^−1^ H_3_BO3, 10 mgL^−1^ FeSO_4_ · 7 H_2_O).

A shake flask preculture (150 mL) was started from a minimal medium working cell bank (8.5 mL) with a glucose concentration of 5 gL^−1^. At mid-exponential growth phase, the preculture was used to inoculate the bioreactor (KLF 3.7 L, Ser. No. 10819, Bioengineering AG, Wald, Switzerland) to reach a final working volume of 1.5 L. Before inoculation, the cultivation conditions were set to 30°C, a stirrer speed of 700 rpm, a pressure of 0.5 bar and an aeration of 2 Lmin^−1^ sterile filtered ambient air. The pH was set and maintained at pH 7 with 25% (v/v) NH_4_OH. Exhaust gas composition (Blue Sense CO_2_ and O_2_, (DCP-CO2 DCP-02, Blue Sense gas sensor GmbH, Herten, Germany), dissolved oxygen and pH in the liquid phase (Ingold, Mettler Toledo GmbH, Giessen, Germany) were monitored online. After glucose depletion, the batch cultivation was continued as a chemostat. At steady state conditions, the dilution rate equals the specific growth rate μ in a chemostat set-up. Each dilution rate (and therefore growth rate) and environmental condition was kept for 5 residence times. The dilution rate was adjusted by feeding at a defined flow rate. Weight gain of the reactor was monitored and a harvest pump was started at a weight gain of 10 g. Additionally, the dilution rate was checked manually by measuring the mass of the harvest outflow within a timespan of one hour before sampling. Steady state was evaluated online via exhaust air analysis. Chemostat cultivations were performed in three individual biological replicates.

### Determination of the adenylate energy charge

The adenylate energy charge (AEC) value mirrors the cellular energy status (Atkinson and Walton [1967]) and can be assessed as follows: Biocatalytic reactions inside the cells were stopped with 35% (w/v) HClO_4_. 4 mL biosuspension was taken directly into 1 mL of precooled (−20°C) HClO_4_ solution on ice and mixed immediately (Theobald et al. [1997]). The sample was shaken at 4°C for 15 min in an overhead rotation shaker. Afterwards, the solution was neutralized on ice by fast addition of 1 mL 1 M K_2_HPO_4_ and 0.9 mL 5 M KOH (Buchholz et al. [2001]). The neutral solution was centrifuged at 4°C and 4,000×g for 10 min to remove cell debris, precipitated protein and potassium perchlorate. The supernatant was kept at −20°C for batch high pressure liquid chromatography (HPLC) measurements. At each sampling time, the biosuspension sample and a filtrated sample without cells was treated according to the above described procedure.

Nucleotide analysis was performed by reversed phase ion pair HPLC (Theobald et al. [1997]). The HPLC system (Agilent Technologies, Waldbronn, Germany) consisted of an Agilent 1200 series autosampler, an Agilent 1200 series Binary Pump SL, an Agilent 1200 series thermostated column compartment, and an Agilent 1200 series diode array detector set at 260 and 340 nm. The nucleotides were separated and quantified on an RP-C-18 column that was combined with a guard column (Supelcosil LC-18-T; 15 cm × 4.6 mm, 3 μm packing and Supelguard LC-18-T replacement cartridges, 2 cm; Supelco, Bellefonte, USA) at a flow rate of 1 ml/min. A gradient elution method (Cserjan-Puschmann et al. [1999]) was adapted and performed with two mobile phases, buffer A (0.1 M KH_2_PO_4_/K_2_HPO_4_, with 4 mM tetrabutylammonium sulfate and 0.5% (v/v) methanol, pH 6.0) and (ii) solvent B (70% (v/v) buffer A and 30% (v/v) methanol, pH 7.2). The following gradient programs were implemented: 100% (v/v) buffer A from 0 min to 3.5 min, increased to 100% (v/v) B until 43.5 min, remaining at 100% (v/v) B until 51 min, decreased to 100% (v/v) A until 56 min and remaining at 100% (v/v) A until 66 min.

The AEC is calculated according to Atkinson and Walton ([1967]):

(1) $$\mathrm{AEC}=\frac{\left[\mathrm{ATP}\right]+0.5\cdot \left[\mathrm{ADP}\right]}{\left[\mathrm{AMP}\right]+\left[\mathrm{ADP}\right]+\left[\mathrm{ATP}\right]}$$

### Sample preparation and staining for flow cytometry

Samples for flow cytometry were washed with PBS, resuspended in cryo-protective solution (15% (v/v) glycerol in PBS according to Jahn et al. ([2013])) and stored at −20°C.

Deep-frozen cell samples were thawed on ice and centrifuged for 2 min at 8,000×g and 4°C to remove the cryo-protective solution. The supernatant was discarded, the cells were resuspended in ice cold PBS and adjusted to an optical density of OD_600nm_ = 0.05 in 2 mL volume. For DNA staining, the cells were centrifuged, taken up in 1 mL permeabilization buffer (0.1 M citric acid, 5 gL^−1^ Tween 20), incubated for 10 min on ice, centrifuged again and the supernatant was removed. Finally, cells were resuspended in 2 mL ice cold staining buffer (0.68 μM DAPI, 0.1 M Na_2_HPO_4_), filtered through a Partec CellTrics mesh (Partec, Germany) with 30 μm pore size and stored on ice until analysis.

### Flow cytometry and cell sorting

Flow cytometry was performed using biological duplicates. For each biological replicate two technical replicates were investigated using a MoFlo cell sorter (Beckman-Coulter, USA) as described before (Jahn et al. [2013]; Jehmlich et al. [2010]). Forward scatter (FSC) and side scatter signals (SSC) were acquired using blue laser excitation (488 nm, 400 mW) and a bandpass filter of 488/10 nm together with a neutral density filter of 2.0 for emission. The DAPI fluorescence was recorded using a multi-line UV laser for excitation (333–365 nm, 100 mW) and a bandpass filter of 450 ± 30 nm for emission. The datasets were annotated according to the miFlowCyt standard (Lee et al. [2008]) and are publicly available on the FlowRepository database (Spidlen et al. [2012]). Cells were sorted at the most accurate mode (single cell, one drop) with a sorting speed of 4,000 s^−1^ and a sample chamber cooled to 4°C. For cell sorting a total number of 5 × 10^6^ cells per replicate were directly sorted on a filter well plate (LoProdyne™ membrane with 0.45 μm pore size, Nunc, Germany) and the residual buffer was constantly drawn off by an exhaust pump. After sorting, the filter membrane was washed three times with 200 μL PBS, air dried and stored at −20°C for further analysis.

### Identification of proteins by LC-MS-MS

For quantitative proteomics, the filter membrane was cut into smaller pieces and treated by trypsin for whole cell proteolytic digestion as described in Jahn et al. ([2013]). The obtained peptide solution was purified using the ZipTip protocol (Millipore, USA), dried in a vacuum concentrator at 30°C and finally taken up in 20 μL 0.1% (w/v) formic acid. The solution was separated by nano-ultra performance liquid chromatography and measured by an LTQ Orbitrap XL (Thermo Fisher Scientific, Germany) as described in Jahn et al. ([2013]).

### Data analysis

Mass spectra were analyzed by MaxQuant v1.2.2.5 (Cox and Mann [2008]) for protein identification and label-free quantification with the genome database of *P. putida* KT2440 and the settings given in Jahn et al. ([2013]). The label-free quantification (LFQ) values were used for further data analysis and can be found in the Additional file 1. The mean, standard deviation and relative quantity of replicates in relation to the reference population (RP, μ = 0.2 h^−1^, mean of two biological replicates) was calculated. The RP was sorted in order to exclude influences of the sorting procedure on the proteomic content. Unsorted cells of the 0.2 h^−1^ grown population were used as an unaffected control population (CP). Student's *t*-test was performed for significance testing (*p* < 0.05) of single proteins. Proteins were annotated using COG (clusters of orthologous groups) (Tatusov et al. [1997]) and clustered in two hierarchical levels of metabolic pathways (‘metabolism’, ‘pathway’). Protein clusters were tested for significant changes using the R Bioconductor (www.bioconductor.org) packages GAGE (Luo et al. [2009]) and GlobalTest (Goeman et al. [2006]), setting *p* < 0.05 and a relative fold change (FC) of 1.5 (log_2_ FC > 0.58) as thresholds. Hierarchical groups were visualized using a color-coded circular treemap (Jahn et al. [2013]).

## Results

**Figure 2:**
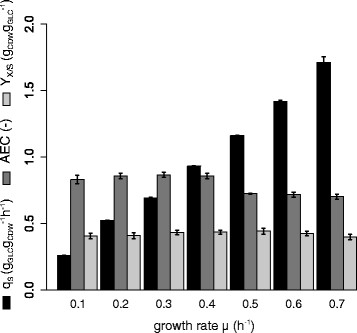
**Summary of the physiological state of the average population.** The specific glucose uptake rate (q_s_, g_GLC_g_CDW_h^−1^, black bars), the adenylate energy charge (AEC, dark grey bars) and the biomass yield (Y_x/s_, g_CDW_g_GLC_^−1^, light grey bars) were measured at steady state conditions for different growth rates μ (h^−1^). The growth rate was stepwise increased until a wash-out of the cells was monitored. Concentrations of cell dry weight (CDW), glucose (GLC) and the AEC were measured offline, sampling after 5 residence times of one specific growth rate (0.1 ≤ μ (h^−1^) ≤ 0.7). Error bars show the standard deviation between three biological replicate cultivations.

Subpopulation dynamics of *P. putida* KT2440 were analyzed in a wide range from slow growth rates starting at μ = 0.1 h^−1^ to high growth rates of up to μ = 0.7 h^−1^. At growth rates higher than μ = 0.7 h^−1^, wash out of the culture was observed, meaning that the maximal growth rate was exceeded and cells could not reproduce fast enough to keep the population density constant. For this reason, μ = 0.7 h^−1^ was the highest growth rate investigated in this study. The physiological and the energetic state of the averaged cell population was analyzed by biomass/substrate yield (Y_x/s_), biomass specific substrate uptake rates (q_s_), and adenylate energy charge measurements (AEC), each measured at steady state growth conditions (Figure 2). Observed stable carbon dioxide emission rates served as the criterion to qualify the achievement of steady-state cultivation conditions.

The yield of biomass on glucose increased gradually by 10% from μ = 0.1 h^−1^ to μ = 0.5 h^−1^. Further rise of the growth rate resulted in yield reductions, returning to the level at μ = 0.1 h^−1^ (−10%). The energetic capacity of the cells can be estimated via AEC, taking the relative contribution of all three phosphorylated forms of adenine into account. The AEC was found to be stable with increasing growth rate until μ = 0.4 h^−1^. Further increasing the growth rate resulted in a reduction of the AEC level by – 18% (*p*-value < 0.01), which was almost the same at maximum growth, still staying in the range of expected physiological levels. The specific glucose uptake rate q_s_ was increasing linearly with increasing growth rate.

**Figure 3:**
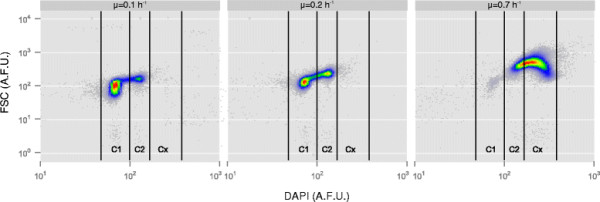
**Dot plots of DNA content (DAPI, in arbitrary fluorescence units (A.F.U.)) versus forward scatter (FSC, in A.F.U.) at different growth rates 0.1 h**^**−1**^**, 0.2 h**^**−1**^**and 0.7 h**^**−1**^**.** The dataset of the biological replicate can be found in the Additional file 2: Figure S1. Cells of *P. putida* KT2440 grown at steady state conditions in chemostats were stained with DAPI and analyzed by flow cytometry. The DNA content and the forward scatter increased with increasing growth rate. The indicated gates (C1, C2, Cx) were used for sorting 5x10^6^ cells per subpopulation for further mass spectrometric analysis.

To be able to distinguish between subpopulations, flow cytometry was proven to be a suitable tool shedding light on the dynamics of single cells within a heterogeneous microbial population (Cooper [1991]; Müller and Babel [2003]; Shapiro [2000]; Skarstad et al. [1985]). Here, the DNA content was monitored via flow cytometry in addition to forward scattering (FSC) giving relative information about cell size (Müller and Nebe-Von-Caron [2010]) (Figure 3). The dataset of the biological replicate can be found in the Additional file 2: Figure S1. The subpopulation analysis revealed that the major differential parameter was the alteration of DNA content as distinguished by flow cytometry. Three subpopulations could be identified in total: cells containing a single chromosome equivalent (C1), two chromosome equivalents (C2) and cells with more than two chromosome equivalents (Cx) (Figure 3). Population composition with respect to DNA content varied clearly as a function of growth rates. At μ = 0.1 h^−1^, 82.0 ± 0.3% of cells contained a single chromosome equivalent, while only 18.0 ± 0.2% contained a double chromosome equivalent content. No Cx subpopulation could be detected. On the contrary, at the high growth rate of μ = 0.7 h^−1^ only 1.4 ± 0.8% of cells belonged to the C1 subpopulation, 16.1 ± 0.1% of cells contained a double chromosome content and 82.5 ± 1.0% more than double.

To investigate whether subpopulations with different DNA content show physiological differences as well, we sorted the cell population at three growth rates (0.1 h^−1^, 0.2 h^−1^ and 0.7 h^−1^) into subpopulations containing single (C1), double (C2) or more than double chromosome content (Cx) aiming to analyze their proteome profile as the basis of their phenotype. In total, 677 unique proteins could be detected. 351 proteins were found in at least one replicate of all subpopulations and 245 proteins were found across all replicates. 707 different functions of 647 unique proteins were annotated using the database of clusters of orthologous groups (COG) (Tatusov et al. [1997]) (Additional file 2: Figure S2). 95.2% of the non-sorted control population (CP) proteome could be found in the reference population (RP) proteome without significant changes, indicating only a small influence of cell sorting on protein recovery and confirming the quality of the analysis.

Significant changes in protein quantity were defined by exceeding a threshold of more than 1.5 fold change (FC) in combination with a *p*-value < 0.05 (Student's *t*-test). Changes in metabolic pathways were detected using GAGE and GlobalTest gene set analysis (Goeman et al. [2006]; Luo et al. [2009]) applying the same significance filter as for the individual proteins.

**Figure 4:**
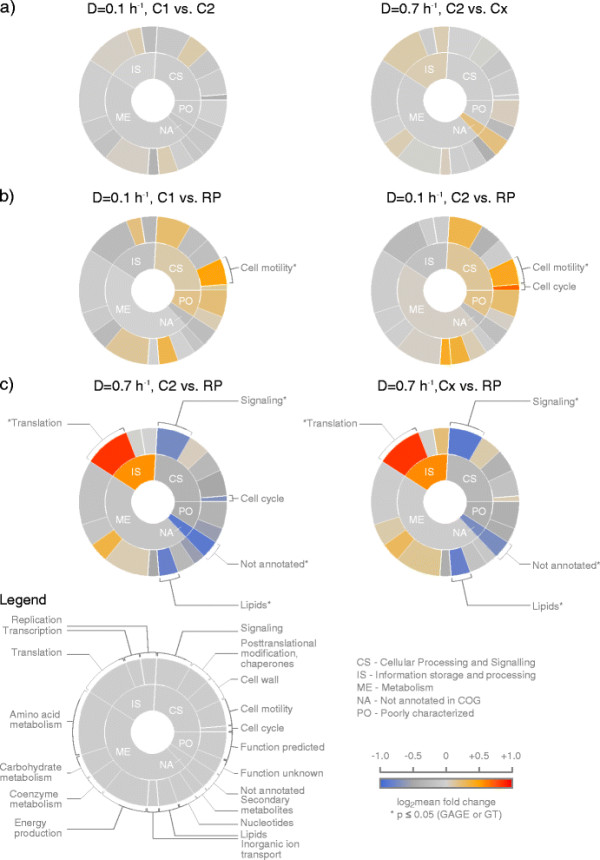
**Circular treemaps visualizing differentially expressed functional protein categories.** Proteins detected by mass spectrometry were clustered according to their pathway annotation in COG covering two levels of specificity (Tatusov et al. [1997]). The size of a sector is proportional to the number of proteins found in one specific pathway in relation to the total protein number. The color code represents the log_2_ mean fold change (log_2_ FC) of protein quantity in one pathway. The color blue codes for an underrepresentation, red for an overrepresentation of the proteins in a pathway compared to the reference population (RP, μ = 0.2 h^−1^). Pathways with a fold change in the range log_2_ FC < −0.58 and log_2_ FC > 0.58 are labeled with the respective pathway name. Pathways that were significantly changed using GAGE (Luo et al. [2009]) and Globaltest (Goeman et al. [2006]) gene set analysis are additionally marked (*). **a.** Comparison of the subpopulations C1/C2 and C2/Cx at growth rates 0.1 h^−1^ and 0.7 h^−1^, respectively. **b.** Comparison of the subpopulations C1 and C2 at μ = 0.1 h^−1^ with RP. **c.** Comparison of the subpopulations C2 and Cx at μ = 0.7 h^−1^ with RP.

As a result, at any given growth rate, the proteomic patterns of the subpopulations did not differ significantly from each other (Figure 4a). When looking at single proteins, only three were detected that comprised significantly different levels between subpopulations at growth rate μ = 0.1 h^−1^ and μ = 0.7 h^−1^, respectively. The abundance of cell division protein FtsZ was found to be 3.6 fold lower in subpopulation C1 in contrast to C2. FtsZ is a bacterial tubulin homologue self-assembling into a ring at mid-cell level and localizing the bacterial divisome machinery (Adams and Errington [2009]; Weart et al. [2007]). The two other proteins were the molecular chaperone GroEL (FC 1.7) and a P-47-like protein (PP_2007, FC 2.4). Also at high growth rate of μ = 0.7 h^−1^, only three proteins, the translocation protein TolB (FC 1.8), the NADH dehydrogenase subunit G (PP_4124, FC 1.51) and a succinyldiaminopimelate transaminase (PP_1588, FC 0.26) showed significant differences between the subpopulations C2 and Cx. Surprisingly, no changes in metabolic pathways could be found between subpopulations at any given growth rate.

**Figure 5:**
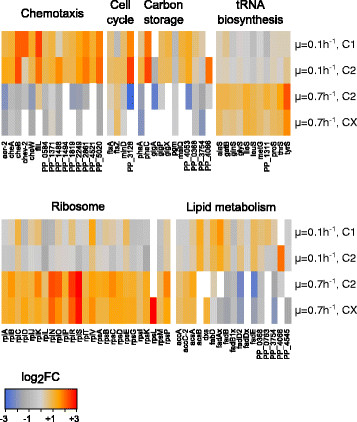
**Heatmaps of metabolic pathways of special interest.** The log_2_ fold changes of annotated proteins are visualized ranging from blue (low abundance) to red (high abundance). A detailed annotation of the protein names can be found in the Additional file 1. One line of the heatmap represents the different subpopulations (C1, C2 and Cx) at different growth rates (μ = 0.1 h^−1^, μ = 0.7 h^−1^). Proteins of the specific pathways are shown column-wise.

Comparing the subpopulations of different growth rates with RP, biologically significant differences were detectable as tested by gene set analysis (GAGE (Luo et al. [2009]) and Globaltest (Goeman et al. [2006])) (Figure 4b and 4c). At μ = 0.1 h^−1^, subpopulations C1 and C2 showed higher abundance of proteins related to ‘cell motility’, and proteins involved in ‘cell cycle control, cell division and chromosome partitioning’ (cell cycle) were additionally highly abundant in subpopulation C2. Apart from COG annotated pathways, several proteins connected to carbon storage were found to be significantly changed (Figure 5). Mirroring low q_S_ at slow growth compared to moderate growth, four main signaling proteins in chemotaxis (CheA, CheB, CheW, CheV) as well as 6 methyl accepting chemotaxis transducers were significantly increased. Furthermore, the low abundance of glycogen synthesis proteins (GlgA, Pgm) and the high abundance of glycogen hydrolysis proteins (GlgX, GlgP) could be seen together with an increase of proteins involved in PHA production (PhaA, PhaC).

In contrast, subpopulations C2 and Cx of fast growing cells (μ = 0.7 h^−1^) revealed higher presence of proteins grouped in the pathway ‘Translation, ribosomal structure and biogenesis’ (Translation), while proteins of 'Signal transduction mechanisms’ (Signaling) and ‘Lipid transport and metabolism’ (Lipids), were significantly underrepresented. The faster growth was reflected in proteins related to translation and therefore protein production. Here, 11 tRNA synthetases and 25 ribosomal proteins showed significantly higher abundance. In lipid metabolism, mostly enzymes of beta-oxidation were found in lower presence at fast growth (Figure 5). The supposed down regulation of the ‘Cell Cycle’ (C2 versus Cx) was mainly due to the single protein change of the poorly characterized PP_3128.

In summary, the proteome of cells differing in DNA content but of identical growth rate was highly similar, whereas the proteome of cells cultivated at different growth rates was significantly diverging in particular pathways.

## Discussion

Considering the influence of different growth rates on the population, proteome analysis revealed that slow growth triggered starvation response, while fast growing cells displayed accelerated protein synthesis and alleviated stress physiology. In slowly growing cells, proteins connected to PHA synthesis and glycerol hydrolysis were amplified, indicating higher PHA carbon storage activity. Additionally, these cells showed protein patterns anticipating increased motility and chemotaxis response. Notably, low q_S_ values of slowly growing cells (μ = 0.1 h^−1^) were not reflected on the energetic state of the population. AEC values did not differ significantly between slow and moderate growth rates of 0.1 h^−1^ and 0.4 h^−1^, respectively. Chemotaxis and cellular motility as a response to carbon-poor conditions are well-known phenomena in natural environments (Harshey [2003]; Soutourina and Bertin [2003]). Our observations in slowly growing cells are in agreement with findings of transcriptome studies in ‘average populations’ of other species. For instance, studies in *E. coli* showed higher expression of genes involved in motility at slower growth rates in direct comparison to faster growth conditions (Nahku et al. [2010]) and studies in *Saccharomyces cerevisiae* showed significant amplification of carbon storage metabolism at slow growth (François and Parrou [2001]).

Fast growing cells were obviously investing resources in proteins involved or related to the translation machinery. Multiple ribosomal proteins as well as tRNA synthetases were highly abundant fostering protein/biomass production (Figure 5). This finding is also in agreement with observations in eukaryotes like *S. cerevisiae* (Rebnegger et al. [2014]) and prokaryotes such as *Salmonella typhimurium* (Schaechter et al. [1958]). Additionally, proteins of typical carbon storage pathways, e.g. PHA synthesis, were less abundant in *P. putida* KT2440. Proteins of lipid biosynthesis, especially involved in beta oxidation were also lowered in fast growing cells compared to RP. This observation is in agreement with the lower abundance of the PHA synthesis proteins, as the beta oxidation provides precursors for this pathway (Aldor and Keasling [2003]).

To our surprise, the almost 6.5-fold increase of the specific glucose uptake rate with increasing growth rate (Figure 2), was not mirrored by major changes among proteins involved in carbohydrate and energy metabolism.

Notably, relative changes of protein quantity can be elucidated with the method applied here. Absolute changes per cell, dependent on the growth rate were not measured with the applied workflow, as it was first shown for the sum of proteins by Schaechter et al. ([1958]). Their pioneering studies described an exponential increase in protein, DNA and RNA contents and therefore, cell size with increasing growth rates (Bremer and Dennis [1996]; Maaløe and Kjeldgaard [1966]; Schaechter et al. [1958]). In our study, the relative cell size estimation was acquired using FSC. In accordance to various other cell cycle analyses, the FSC increased with increasing growth rates (Donachie [1968]; Hewitt et al. [1999]; Neumeyer et al. [2013]; Skarstad et al. [1983]) (Figure 3). Following the rational of Schaechter et al. ([1958]), this phenomenon reflects increasing protein contents per cell. We presume that the increased amount of cellular glucose uptake is proportional to the elevated production of proteins, thus increasing absolute protein quantity but leaving relative quantity unchanged.

Studying the putative impact of growth rate and cell cycle stage on the functional diversity of a population, the growth rate is obviously a major determinant for cellular protein composition, as found in our chemostat studies. Growth and cell cycle were clearly linked, but subpopulations showing different DNA content showed only small differences in cellular physiology at the same growth rate. The detection of FtsZ in a significant higher abundance in the C2 subpopulation, which is preparing for division after finishing replication, is in agreement with its assigned function as a proposed diffusible factor (Teather et al. [1974]) initiating cell division (Chien et al. [2012]). Despite this cell cycle related finding, subpopulations showed almost identical protein patterns irrespective of cell sizes, anticipated protein mass (Lindmo [1982]; Rønning et al. [1979]) and DNA content.

Surprisingly, no signs for a specialization of cells in different cell stages for e.g. carbon storage or protein production/growth could be observed that could support the hypothesis of shared tasks of subpopulations in B- and pre-D/D-phases during the cell cycle. This result is remarkable: subpopulations distinguished by DNA content appear to be physiologically highly similar provided that the growth rate is the same.

Although we are aware that subpopulations do not mirror single cell proteome compositions, the high resemblance of the subpopulations proteome patterns at the various growth rates point to their nearly identical physiological state.

One may argue whether this finding was influenced by the operation mode ‘chemostat’. We identified the high similarity among subpopulations by installing distinct growth rates, because superimposing impacts in classical (fed-) batch fermentations would have prevented the unequivocal growth-to-subpopulation analysis. However, the chemostat approach might have excluded the detection of subpopulations with different protein contents because this ‘growth rate filter’ was installed. Assuming that cells aim to grow with the least energetic burden as possible, cellular protein compositions should be optimized at a given growth rate. Therefore, it could not be excluded, that subpopulations showing different protein patterns may have existed, but were washed-out because they could not achieve the required growth rate. While the latter demands for further in-depth analysis, the determining impact of growth on cell cycle and subpopulations is clearly visible. It gives rise to the assumption that the cell cycle itself has a minor impact on population heterogeneity under the conditions tested.

## Competing interests

The authors declare that they have no competing interests.

## Authors’ contributions

SL carried out the chemostat cultivations, analysed the datasets and drafted the manuscript. MJ carried out the flow cytometry measurements, analysed the datasets and corrected the manuscript. JS and MB performed the mass spectrometry measurements. SM and RT conceived the study and corrected the manuscript. All authors read and approved the final manuscript.

## Additional files

## Supplementary Material

Additional file 1: Dataset of the label-free quantification (LFQ) values that were used for further analysis of differences in protein pattern. Click here for file

Additional file 2: Figure S1. Replicate dataset of dot plots of DNA content and **Figure S2**: Overview of the total protein detection and protein annotation. Click here for file

## References

[B1] Ackermann J-U Müller S Lösche A Bley T Babel W *Methylobacterium rhodesianum* cells tend to double the DNA content under growth limitations and accumulate PHB J Biotechnol 1995 39 9 20 10.1016/0168-1656(94)00138-3

[B2] Adams DW Errington J Bacterial cell division: assembly, maintenance and disassembly of the Z ring Nat Rev Microbiol 2009 7 642 653 10.1038/nrmicro2198 19680248

[B3] Aldor IS Keasling JD Process design for microbial plastic factories: metabolic engineering of polyhydroxyalkanoates Curr Opin Biotechnol 2003 14 475 483 10.1016/j.copbio.2003.09.002 14580576

[B4] Atkinson DE Walton GM Adenosine triphosphate conservation in metabolic regulation: rat liver citrate cleavage enzyme J Biol Chem 1967 242 3239 3241 6027798

[B5] Avery SV Microbial cell individuality and the underlying sources of heterogeneity Nat Rev Microbiol 2006 4 577 587 10.1038/nrmicro1460 16845428

[B6] Bagdasarian M Lurz R Rückert B Franklin F Bagdasarian M Frey J Timmis K Specific-purpose plasmid cloning vectors II. Broad host range, high copy number, RSF 1010-derived vectors, and a host-vector system for gene cloning in *Pseudomonas* Gene 1981 16 237 247 10.1016/0378-1119(81)90080-9 6282695

[B7] Blank LM Ionidis G Ebert BE Bühler B Schmid A Metabolic response of *Pseudomonas putida* during redox biocatalysis in the presence of a second octanol phase FEBS J 2008 275 5173 5190 10.1111/j.1742-4658.2008.06648.x 18803670

[B8] Bley T State-structure models—A base for efficient control of fermentation processes Biotechnol Adv 1990 8 233 259 10.1016/0734-9750(90)90014-3 14545912

[B9] Brehm-Stecher BF Johnson EA Single-cell microbiology: tools, technologies, and applications Microbiol Mol Biol Rev 2004 68 538 559 10.1128/MMBR.68.3.538-559.2004 15353569 PMC515252

[B10] Bremer H Dennis PP Modulation of chemical composition and other parameters of the cell by growth rate 1996 10.1128/ecosal.5.2.3 26443740

[B11] Buchholz A Takors R Wandrey C Quantification of intracellular metabolites in *Escherichia coli* K12 using liquid chromatographic-electrospray ionization tandem mass spectrometric techniques Anal Biochem 2001 295 129 137 10.1006/abio.2001.5183 11488613

[B12] Carlquist M Fernandes R Helmark S Heins A-L Lundin L Sorensen S Gernaey K Lantz A Physiological heterogeneities in microbial populations and implications for physical stress tolerance Microb Cell Fact 2012 11 94 10.1186/1475-2859-11-94 22799461 PMC3443036

[B13] Chien A-C Hill NS Levin PA Cell size control in bacteria Curr Biol 2012 22 R340 R349 10.1016/j.cub.2012.02.032 22575476 PMC3350639

[B14] Cooper S Bacterial Growth and Division: Biochemistry and Regulation of Prokaryotic and Eukaryotic Division Cycles 1991 Academic Press, Inc., San Diego, CA

[B15] Cox J Mann M MaxQuant enables high peptide identification rates, individualized ppb-range mass accuracies and proteome-wide protein quantification Nat Biotechnol 2008 26 1367 1372 10.1038/nbt.1511 19029910

[B16] Cserjan-Puschmann M Kramer W Duerrschmid E Striedner G Bayer K Metabolic approaches for the optimisation of recombinant fermentation processes Appl Microbiol Biotechnol 1999 53 43 50 10.1007/s002530051612 10645624

[B17] Delvigne F Goffin P Microbial heterogeneity affects bioprocess robustness: Dynamic single-cell analysis contributes to understanding of microbial populations Biotechnol J 2013 9 61 72 10.1002/biot.201300119 24408611

[B18] Donachie W Relationship between cell size and time of initiation of DNA replication Nature 1968 219 1077 1079 10.1038/2191077a0 4876941

[B19] François J Parrou JL Reserve carbohydrates metabolism in the yeast *Saccharomyces cerevisiae* FEMS Microbiol Rev 2001 25 125 145 10.1016/S0168-6445(00)00059-0 11152943

[B20] Goeman JJ van De Geer SA van Houwelingen HC Testing against a high dimensional alternative J R Stat Soc Ser B (Stat Method) 2006 68 477 493 10.1111/j.1467-9868.2006.00551.x

[B21] Harshey RM Bacterial motility on a surface: many ways to a common goal Annu Rev Microbiol 2003 57 249 273 10.1146/annurev.micro.57.030502.091014 14527279

[B22] Hewitt CJ Nebe-von-Caron G Nienow AW McFarlane CM The use of multi-parameter flow cytometry to compare the physiological response of *Escherichia coli* W3110 to glucose limitation during batch, fed-batch and continuous culture cultivations J Biotechnol 1999 75 251 264 10.1016/S0168-1656(99)00168-6 10553662

[B23] Jahn M Seifert J Hübschmann T von Bergen M Harms H Müller S Comparison of preservation methods for bacterial cells in cytomics and proteomics JIOMICS 2013 3 25 33

[B24] Jehmlich N Hübschmann T Gesell Salazar M Völker U Benndorf D Müller S von Bergen M Schmidt F Advanced tool for characterization of microbial cultures by combining cytomics and proteomics Appl Microbiol Biotechnol 2010 88 575 584 10.1007/s00253-010-2753-6 20676634

[B25] Lee JA Spidlen J Boyce K Cai J Crosbie N Dalphin M Furlong J Gasparetto M Goldberg M Goralczyk EM Hyun B Jansen K Kollmann T Kong M Leif R McWeeney S Moloshok TD Moore W Nolan G Nolan J Nikolich-Zugich J Parrish D Purcell B Qian Y Selvaraj B Smith C Tchuvatkina O Wertheimer A Wilkinson P Wilson C Wood J Zigon R Scheuermann RH Brinkman RR MIFlowCyt: The minimum information about a flow cytometry experiment Cytom Part A 2008 73A 926 930 10.1002/cyto.a.20623 PMC2773297 18752282

[B26] Lencastre Fernandes R Nierychlo M Lundin L Pedersen AE Puentes Tellez PE Dutta A Carlquist M Bolic A Schäpper D Brunetti AC Helmark S Heins A-L Jensen AD Nopens I Rottwitt K Szita N van Elsas JD Nielsen PH Martinussen J Sørensen SJ Lantz AE Gernaey KV Experimental methods and modeling techniques for description of cell population heterogeneity Biotechnol Adv 2011 29 575 599 10.1016/j.biotechadv.2011.03.007 21540103

[B27] Lindmo T Kinetics of protein and DNA synthesis studied by mathematical modelling of flow cytometric protein and DNA histograms Cell Tissue Kinet 1982 15 197 211 7066960 10.1111/j.1365-2184.1982.tb01038.x

[B28] Luo W Friedman M Shedden K Hankenson K Woolf P GAGE: generally applicable gene set enrichment for pathway analysis BMC Bioinformatics 2009 10 161 10.1186/1471-2105-10-161 19473525 PMC2696452

[B29] Maaløe O Kjeldgaard NO Control of macromolecular synthesis: a study of DNA, RNA, and protein synthesis in bacteria 1966 W.A. Benjamin, Inc., New York, NY

[B30] Martins dos Santos VAP Heim S Moore ERB Strätz M Timmis KN Insights into the genomic basis of niche specificity of *Pseudomonas putida* KT2440 Environ Microbiol 2004 6 1264 1286 10.1111/j.1462-2920.2004.00734.x 15560824

[B31] Meijnen J-P de Winde JH Ruijssenaars HJ Engineering *Pseudomonas putida* S12 for efficient utilization of D-xylose and L-arabinose Appl Environ Microbiol 2008 74 5031 5037 10.1128/AEM.00924-08 18586973 PMC2519266

[B32] Müller S Modes of cytometric bacterial DNA pattern: A tool for pursuing growth Cell Proliferat 2007 40 621 639 10.1111/j.1365-2184.2007.00465.x PMC6496216 17877606

[B33] Müller S Babel W Analysis of bacterial DNA patterns - An approach for controlling biotechnological processes J Microbiol Meth 2003 55 851 858 10.1016/j.mimet.2003.08.003 14607431

[B34] Müller S Nebe-von-Caron G Functional single-cell analyses: Flow cytometry and cell sorting of microbial populations and communities FEMS Microbiol Rev 2010 34 554 587 20337722 10.1111/j.1574-6976.2010.00214.x

[B35] Müller S Harms H Bley T Origin and analysis of microbial population heterogeneity in bioprocesses Curr Opin Biotechnol 2010 21 100 113 10.1016/j.copbio.2010.01.002 20138500

[B36] Nahku R Valgepea K Lahtvee P-J Erm S Abner K Adamberg K Vilu R Specific growth rate dependent transcriptome profiling of *Escherichia coli* K12 MG1655 in accelerostat cultures J Biotechnol 2010 145 60 65 10.1016/j.jbiotec.2009.10.007 19861135

[B37] Nakazawa T Yokota T Benzoate metabolism in *Pseudomonas putida (arvilla)* mt-2: demonstration of two benzoate pathways J Bacteriol 1973 115 262 267 4717515 10.1128/jb.115.1.262-267.1973 PMC246238

[B38] Nelson K Weinel C Paulsen I Dodson R Hilbert H Martins dos Santos V Fouts D Gill S Pop M Holmes M Complete genome sequence and comparative analysis of the metabolically versatile *Pseudomonas putida* KT2440 Environ Microbiol 2002 4 799 808 10.1046/j.1462-2920.2002.00366.x 12534463

[B39] Neumeyer A Hübschmann T Müller S Frunzke J Monitoring of population dynamics of *Corynebacterium glutamicum* by multiparameter flow cytometry Microb Biotechnol 2013 6 157 167 10.1111/1751-7915.12018 23279937 PMC3917458

[B40] Poblete-Castro I Becker J Dohnt K Santos V Wittmann C Industrial biotechnology of *Pseudomonas putida* and related species Appl Microbiol Biotechnol 2012 93 2279 2290 10.1007/s00253-012-3928-0 22350258

[B41] Puchałka J Oberhardt MA Godinho M Bielecka A Regenhardt D Timmis KN Papin JA Martins dos Santos VAP Genome-scale reconstruction and analysis of the *Pseudomonas putida* KT2440 metabolic network facilitates applications in biotechnology PLoS Comp Biol 2008 4 10 e1000210 10.1371/journal.pcbi.1000210 PMC2563689 18974823

[B42] Rebnegger C Graf AB Valli M Steiger MG Gasser B Maurer M Mattanovich D In *Pichia pastoris*, growth rate regulates protein synthesis and secretion, mating and stress response Biotechnol J 2014 9 511 525 10.1002/biot.201300334 24323948 PMC4162992

[B43] Rønning ØW Pettersen EO Seglen PO Protein synthesis and protein degradation through the cell cycle of human NHIK 3025 cells in vitro Exp Cell Res 1979 123 63 72 10.1016/0014-4827(79)90421-X 488184

[B44] Schaechter M Maaløe O Kjeldgaard N Dependency on medium and temperature of cell size and chemical composition during balanced growth of *Salmonella typhimurium* J Gen Microbiol 1958 19 592 606 10.1099/00221287-19-3-592 13611202

[B45] Shapiro HM Microbial analysis at the single-cell level: tasks and techniques J Microbiol Meth 2000 42 3 16 10.1016/S0167-7012(00)00167-6 11000426

[B46] Skarstad K Steen HB Boye E Cell cycle parameters of slowly growing *Escherichia coli* B/r studied by flow cytometry J Bacteriol 1983 154 656 662 6341358 10.1128/jb.154.2.656-662.1983 PMC217513

[B47] Skarstad K Steen HB Boye E *Escherichia coli* DNA distributions measured by flow cytometry and compared with theoretical computer simulations J Bacteriol 1985 163 661 668 3894332 10.1128/jb.163.2.661-668.1985 PMC219173

[B48] Soutourina OA Bertin PN Regulation cascade of flagellar expression in Gram-negative bacteria FEMS Microbiol Rev 2003 27 505 523 10.1016/S0168-6445(03)00064-0 14550943

[B49] Spidlen J Breuer K Rosenberg C Kotecha N Brinkman RR FlowRepository - A resource of annotated flow cytometry datasets associated with peer-reviewed publications Cytom Part A 2012 81 727 731 10.1002/cyto.a.22106 22887982

[B50] Tatusov RL Koonin EV Lipman DJ A genomic perspective on protein families Science 1997 278 631 637 10.1126/science.278.5338.631 9381173

[B51] Teather R Collins J Donachie W Quantal behavior of a diffusible factor which initiates septum formation at potential division sites in *Escherichia coli* J Bacteriol 1974 118 407 413 4597442 10.1128/jb.118.2.407-413.1974 PMC246772

[B52] Theobald U Mailinger W Baltes M Rizzi M Reuss M In vivo analysis of metabolic dynamics in *Saccharomyces cerevisiae*: I. Experimental observations Biotechnol Bioeng 1997 55 305 316 10.1002/(SICI)1097-0290(19970720)55:2<305::AID-BIT8>3.0.CO;2-M 18636489

[B53] Unthan S Grünberger A van Ooyen J Gätgens J Heinrich J Paczia N Wiechert W Kohlheyer D Noack S Beyond growth rate 0.6: What drives *Corynebacterium glutamicum* to higher growth rates in defined medium Biotechnol Bioeng 2014 111 359 371 10.1002/bit.25103 23996851

[B54] Weart R Lee A Chien A-C Haeusser D Hill N Levin P A metabolic sensor governing cell size in bacteria Cell 2007 130 335 347 10.1016/j.cell.2007.05.043 17662947 PMC1971218

[B55] Yuste L Hervás AB Canosa I Tobes R Jiménez JI Nogales J Pérez-Pérez MM Santero E Díaz E Ramos JL De Lorenzo V Rojo F Growth phase-dependent expression of the *Pseudomonas putida* KT2440 transcriptional machinery analysed with a genome-wide DNA microarray Environ Microbiol 2006 8 165 177 10.1111/j.1462-2920.2005.00890.x 16343331

