# Supplementary material for: Subpopulation-proteomics reveal growth rate, but not cell cycling, as a major impact on protein composition in Pseudomonas putida KT2440
Source: AMB Express. 2014 Aug 29;4:71. doi: 10.1186/s13568-014-0071-6 (PMC4230896; doi:10.1186/s13568-014-0071-6)
Supplement: Additional file 2: Figure S1. — Replicate dataset of dot plots of DNA content and Figure S2: Overview of the total protein detection and protein annotation. [file s13568-014-0071-6-S2.pdf]

Supplementary Figures S1 and S2

AMB Express

Subpopulation-proteomics reveal growth rate, but not cell cycling, as a major impact on protein composition in *Pseudomonas putida* KT2440

Sarah Lieder,[a] Michael Jahn,[b] Jana Seifert[c,d], Martin von Bergen[c,e,f], Susann Müller[b], Ralf Takors[a]#

[a] Institute for Biochemical Engineering, University of Stuttgart, Allmandring 31, Stuttgart, Germany

[b] Department of Environmental Microbiology, Helmholtz Centre for Environmental Research—UFZ, Permoserstr. 15, 04318 Leipzig, Germany

[c] Department of Proteomics, Helmholtz Centre for Environmental Research—UFZ, Permoserstr. 15, 04318 Leipzig, Germany

[d] Institute of Animal Nutrition, University of Hohenheim, Emil-Wolff-Straße 8 and 10, 70599 Stuttgart, Germany

[e] Department of Metabolomics, Helmholtz Centre for Environmental Research—UFZ, Permoserstr. 15, 04318 Leipzig, Germany

[f] Department of Biotechnology, Chemistry and Environmental Engineering, University of Aalborg, Sohngaardsholmsvej 49, 9000 Aalborg, Denmark

#Address correspondence to Ralf Takors, takors@ibvt.uni-stuttgart.de, phone +49 711 685 64574, fax +49 711 685 65164

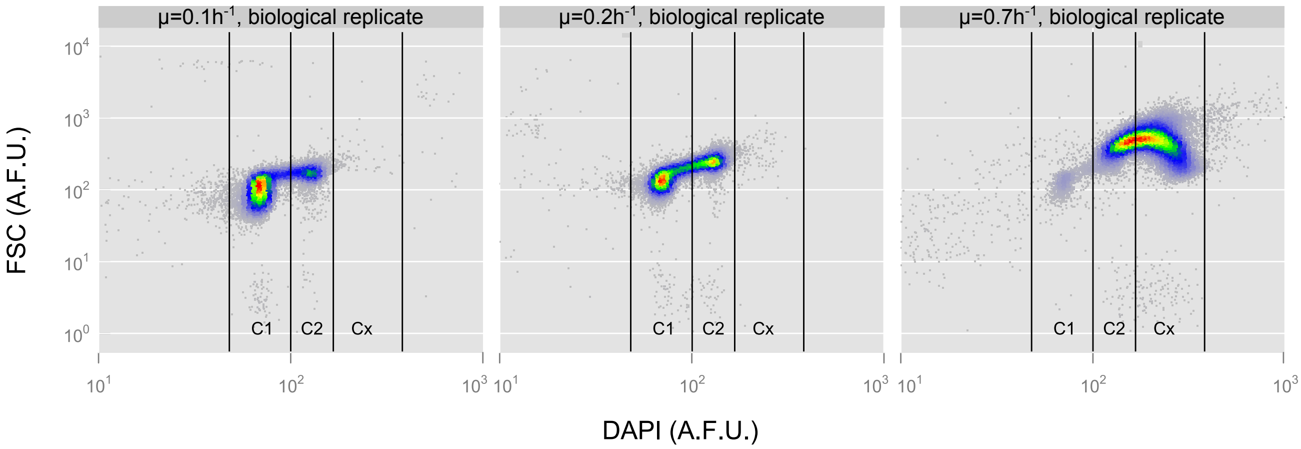

Supplementary Fig. S1 Replicate dataset of dot plots of DNA content (DAPI, in arbitrary fluorescence units (A.F.U.)) versus forward scatter (FSC, in A.F.U.)) at different growth rates 0.1 h^{-1}, 0.2 h^{-1} and 0.7 h^{-1}. Cells of *P. putida* KT2440 grown at steady state conditions in chemostats were stained with DAPI and analyzed by flow cytometry. The DNA content and the forward scatter increased with increasing growth rate. The indicated gates (C1, C2, Cx) were used for sorting $5x10^6$ cells per subpopulation for further mass spectrometric analysis.

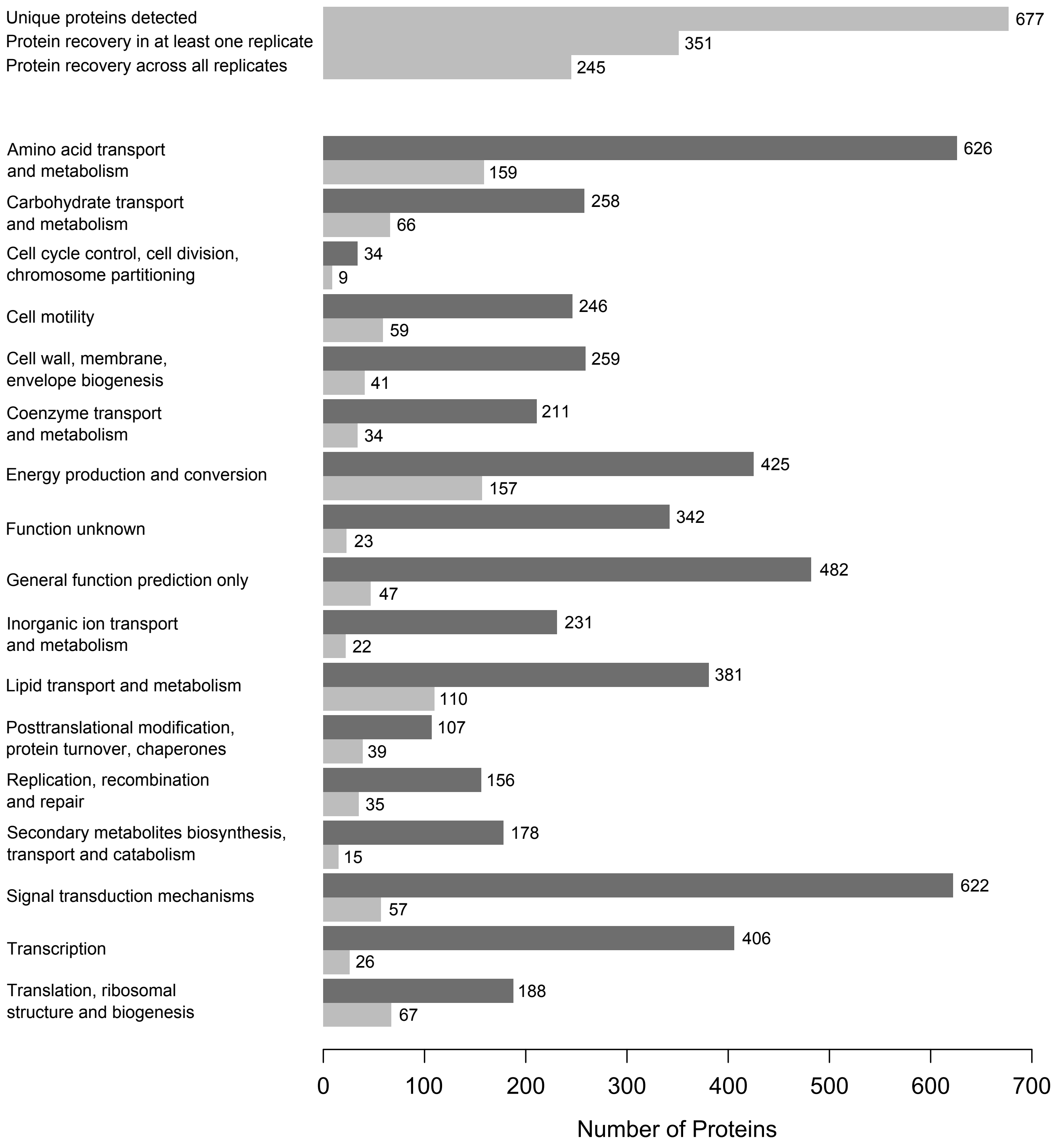

Supplementary Fig. S2 Overview of the total protein detection and protein annotation. Overall, 677 unique proteins were identified, 351 proteins were detected in at least one replicate of all subpopulations and 245 proteins were found across all replicates. Functional annotation was carried out using the COG database (Tatusov et a. 1997). 707 different functions of 647 unique proteins could be annotated into 17 categories. The total number of proteins of *Pseudomonas putida* KT2440 annotated in one specific category (dark grey bars) is compared to the number of proteins recovered in this study (light grey bars).
